# An efficient method for monitoring small bird targets in wetland environments based on object detection

**DOI:** 10.1038/s41598-026-46593-9

**Published:** 2026-04-02

**Authors:** Chuanjun Xing, Chenpeng Qu, Pinghao Zhang, Xiaolin Qin

**Affiliations:** 1 College of Computer Science and Technology, Heilongjiang Institute of Technology, Harbin, 150050 China; 2 College of Computer and Control Engineering, Northeast Forestry University, Harbin, 150040 China; 3 College of Surveying and Mapping Engineering, Heilongjiang Institute of Technology, Harbin, 150050 China; 4 Chengdu Institute of Computer Applications, Chinese Academy of Sciences, University of Chinese Academy of Sciences, Chengdu College, Chengdu, 610213 China

**Keywords:** Detection of wetland birds, Small object detection, YOLOv8, Attention mechanisms, Separable convolutions, Feature integration, Computational biology and bioinformatics, Ecology, Ecology, Environmental sciences

## Abstract

Birds play an essential role in evaluating the health and biodiversity of wetland ecosystems. Due to the complex and diverse wetland environments and the typically small size of birds, existing technologies face issues of low detection accuracy and high miss rates. To address these challenges, this study proposes the RLCB-YOLO model, which is a framework for detecting wetland birds based on YOLOv8n. By combining receptive field attention and coordinate attention, the proposed convolutional modules solve the problem of attention weight sharing and enhance long-range information processing. Additionally, the SPPF-LSKA module is introduced to use long-range dependencies and adaptive scaling, effectively filtering background noise in complex wetland environments. For feature fusion, an improved BiFPN-P2 structure is adopted to facilitate superior cross-scale information interaction. The framework is completed by a content-aware feature reorganization module at the up-sampling stage, ensuring precise focus on the key semantic features of small-scale targets. Experimental results showed that RLCB-YOLO achieves 82.1% mAP@0.5 and 48.6% mAP@0.5:0.95 on a self-built small wetland bird targets dataset, outperforming the baseline YOLOv8n by 3.6% and 2.9%. Furthermore, it outperforms YOLOv8s in overall efficacy while maintaining a reduced parameter count. Visualization analysis further confirms the model’s suitability for engineering applications in ecological monitoring of complex wetland scenes.

## Introduction

Wetlands are one of the most important ecosystems on Earth, playing a crucial role in water conservation, climate regulation, and maintaining biodiversity^1^. However, global climate change and intensified human activities render wetland ecosystems increasingly fragile, facing unprecedented challenges^2^. Birds are important bio-indicators in wetland ecosystems, and their abundance and distribution can provide valuable information on ecosystem condition and biodiversity status^3,4^. To help the preservation of wetland ecosystems, the study focuses on the species abundance estimation and target localization of wetland birds.

Before estimating the number of birds or examining their behavior, it is essential to accurately identify and locate the targets. By improving the ability to detect and locate individual birds from complex wetland scenes, researchers can better monitor habitat use, migration patterns, and species interactions. It offers the information required for ecological assessment and population estimation^5^.

While wetland birds monitoring plays an essential role in ecosystems research, it has long relied on manual methods, including point counts conducted directly in the field by ecologists and ornithologists, as well as static observations using high-power telescopes and telephoto cameras in specific areas^6,7^. These methods require substantial human and material resources and are susceptible to geographical and climatic constraints, resulting in limitations in real-time capability and coverage, thus restricting the accuracy and efficiency of monitoring^8^. Moreover, large-scale manual monitoring activities may cause unnecessary disturbances to sensitive wetland ecosystems^9^. Therefore, developing new technology to enhance monitoring efficiency while minimizing ecological disturbance has become an urgent need for wetland conservation and biodiversity research.

As artificial intelligence technology advances rapidly, its usage across various domains has become more and more extensive^10,11^. Among them, computer vision, as a critical branch of AI, has found extensive applications in animal detection^12–14^. Compared with traditional methods, computer vision techniques provide higher accuracy and efficiency^15–17^. To detect birds in complex urban environments more accurately, Guo et al. proposed a model integrating the Swin Transformer, “Swan-mask R-CNN with SAHI,” which increased the mean average precision by 10%, reaching 74%, compared to traditional Mask R-CNN^18^. Takeki et al. proposed a scale-aware bird detection method that combines deep detectors and semantic segmentation, using ResNet as the detector, FCNs, and DeepLab for semantic segmentation, and integrating detection results with a support vector machine (SVM), thereby enhancing the accuracy of automatic bird detection^19^. Jo et al. employed an improved Inception-v3 model, effectively recognizing birds in dynamic environments through background separation and moving object extraction techniques^20^. These methods have enhanced detection efficiency using convolutional neural networks. However, the high computational resource requirements pose challenges for resource-limited devices.

Since its introduction in 2015, the YOLO algorithm has directly predicted class probabilities and bounding boxes through a single network, approaching the object detection task as a unified regression problem^21^. This approach significantly simplifies the detection process and is widely used in various real-time visual recognition tasks compared to multi-stage convolutional neural networks. Hong et al. utilized aerial photographs collected by UAVs to construct a deep learning-based object detection model^22^. By comparing Faster R-CNN, SSD, and YOLO models, they found that the YOLO model was most advantageous regarding deployment and detection speed. Jiang et al. introduced OD-conv technology to optimize the YOLOv7 model, reducing the model’s parameter count and achieving a bird detection accuracy of mAP 78.42% on power transmission lines^23^. Lei et al. improved YOLOv7 by adding an additional prediction head and integrating the SimAM attention module, achieving an mAP of 67.3% in waterbird monitoring videos^24^. Although this method can improve real-time monitoring efficiency, it still has limitations in detecting dense small targets. Chen et al. combined object detection and multi-object tracking networks, adding three global attention modules (GAM) to YOLOv7 and using the Alpha-IoU loss function for precise bounding box regression, making progress in bird tracking and population counting^25^. However, it still faces limitations in complex backgrounds.

Reviewing recent research, significant achievements in bird detection have been made by introducing deep learning techniques. However, current research lacks models suitable for detecting small bird targets in complex wetland environments. Wetland environments are complex and varied, particularly with variable hydrological conditions, abundant vegetation cover, and diverse lighting conditions^26^. Additionally, birds often appear as small targets in the field of view, especially when they are far from the observer or monitoring equipment, making identification a significant challenge. These factors lead to considerable challenges for reliable monitoring. Moreover, although deep learning methods show great potential in improving recognition accuracy, they usually require large amounts of parameters and computing resources, limiting their feasibility for widespread deployment in resource-constrained applications.

Based on this analysis, this paper proposes the RLCB-YOLO model, which enhances the detection ability of small bird targets in complex wetland scenes while reducing model parameters. This model effectively improves the detection efficiency and accuracy of small bird targets in wetland environments, offering robust support for ecosystem assessment and bird conservation. The primary contributions of this work are summarized as follows:

(1) This study creates a small bird target dataset for wetland scenes that includes a variety of difficult situations, including as high target density, occlusion, and environmental interference. This dataset is useful for model training and testing.

(2) The Receptive-Field Coordinate feature extraction module is introduced. This module uses orientation-sensitive modeling to overcome the feature homogenization caused by parameter sharing in standard convolutions. It enhances the model’s ability to jointly represent local details and global context.

(3) The Large Separable Kernel Attention is adopted to reconstruct the SPPF layer. This captures long-range dependencies while maintaining low computational costs, effectively enhancing the model’s adaptability to complex backgrounds.

(4) In the neck part of the network, an improved BiFPN-P2 structure is used for feature fusion, enhancing the capability to capture lower-level detail information. A content-aware feature reorganization module is optimized the up-sampling process, boosting the model’s ability to reconstruct small target features.

## Materials and methods

### Dataset processing

#### Data acquisition

Research on detecting small bird targets in complex wetland environments remains relatively sparse. In response, a dataset was constructed to solve the challenge of detecting small bird targets in complex wetland environments. On-site fieldwork was carried out at the Sun Island National Wetland Park in Harbin, China, covering several seasons from spring to autumn. Images were captured using smartphones and telephoto cameras to reflect authentic ecological conditions, including natural illumination variations, vegetation occlusion, and water reflections. Keyframes were extracted from fixed wetland surveillance videos at intervals of 1–3 s.

**Fig. 1:**
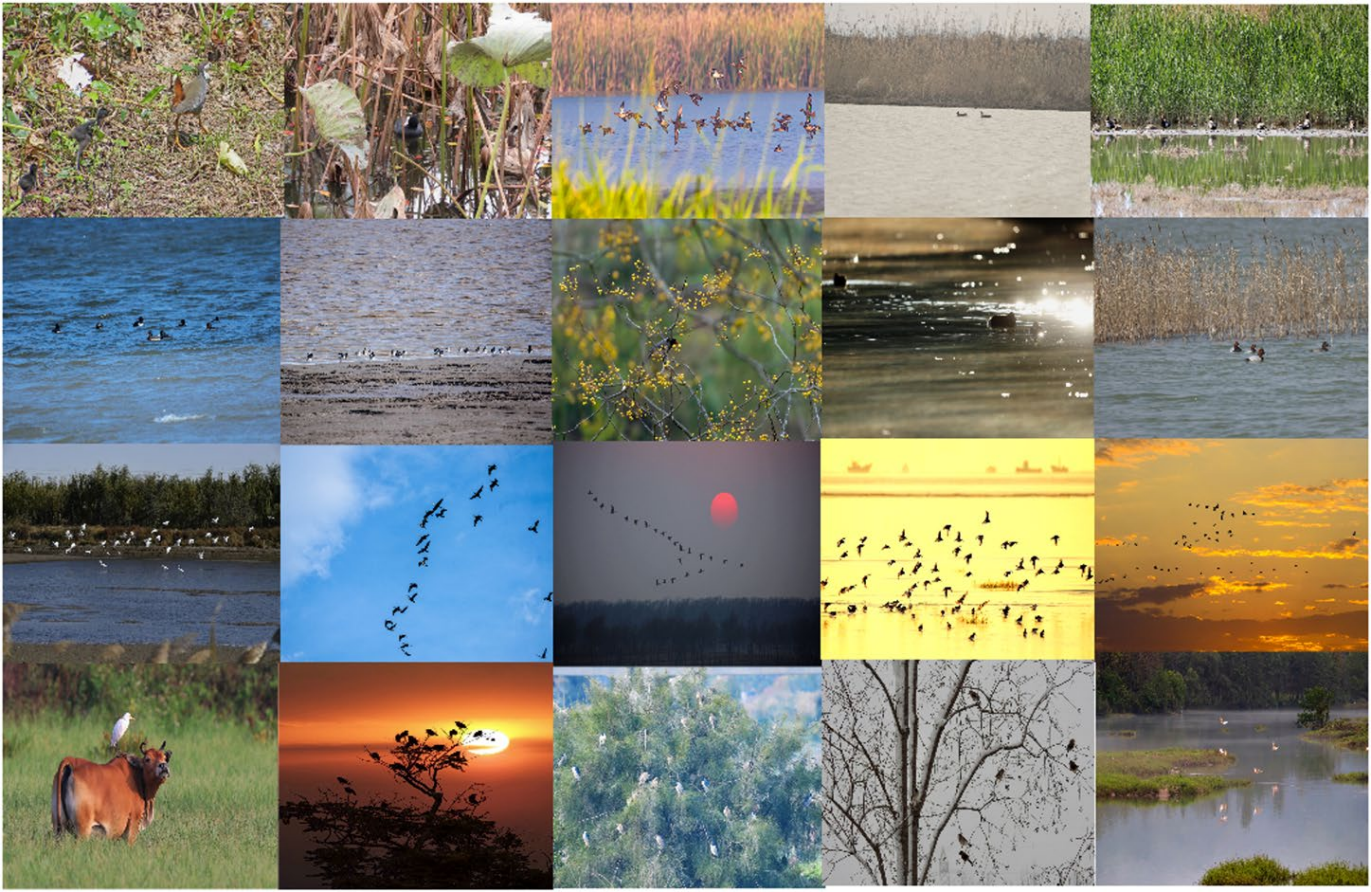
Some samples in the dataset.

During the data screening and cleaning process, we prioritized the retention of samples characterized by small target dimensions (generally occupying less than 10% of the image area), significant background clutter, camouflage, or occlusion. This approach ensures that the dataset accurately reflects the inherent challenges associated with detecting small targets in typical wetland scenes. Figure 1 illustrates relevant scenes from dataset.

#### Data augmentation

**Fig. 2:**
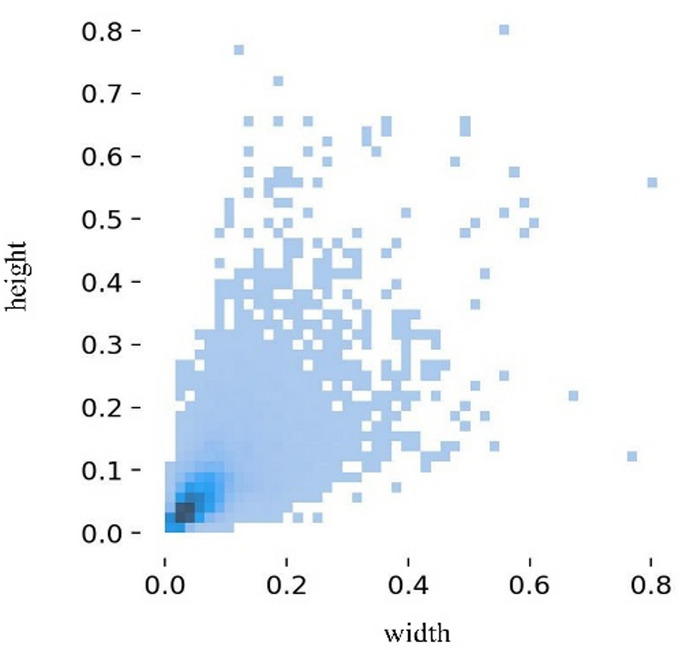
Target box size distribution.

Following a rigorous screening process, the dataset is characterized by a predominance of small-scale avian targets, with the majority of bounding boxes occupying less than 10% of the total image area. Figure 2 illustrates the bounding box size distribution. The horizontal and vertical axes indicate the normalized ratios of the bounding box width and height in connection with the image size, each with values ranging from 0 to 1. The observation that the majority of bounding boxes’ aspect ratios are centered in the lower value range further indicates the dataset’s usual features for small object detection tasks. The final constructed dataset consists of 2,000 images, which were randomly partitioned into training, validation, and testing sets in a 7:2:1 ratio, containing 1,400, 400, and 200 images. The LabelImg tool was used to annotate each picture using the standard YOLO label format.

An offline data augmentation technique was only used on the training set in order to strengthen the model’s robustness and generalization in difficult wetland situations. The validation and test sets remained unaltered to ensure the objectivity and integrity of the performance evaluation. Specifically, two augmented samples were generated offline for each original training image. Each sample underwent a sequential processing pipeline consisting of rotational transformation, random noise injection (randomly selected between Gaussian and salt-and-pepper noise), and brightness adjustment. This approach facilitated the construction of diverse disturbed samples under various parameter combinations. As a result, the training set was increased from 1,400 to 4,200 pictures. By incorporating these imaging perturbations, the model’s adaptability to cluttered backgrounds, small-scale targets, and fluctuating illumination is significantly enhanced, leading to improved training stability and detection reliability. Table 1. 

**Table 1:** Parameter configurations for offline data augmentation.

Method	Parameter settings
Rotation	[−15°~15°]
Noise Injection	(1)Gaussian noise: σ∈[5, 15] (2)Salt pepper noise: density∈[0.002, 0.01]
Brightness Adjustment	[0.7,1.3]

Note: To ensure a balanced representation of offline data augmentation, a 1:1 ratio was maintained between Gaussian and salt-and-pepper noise during the injection process.

**Fig. 3:**
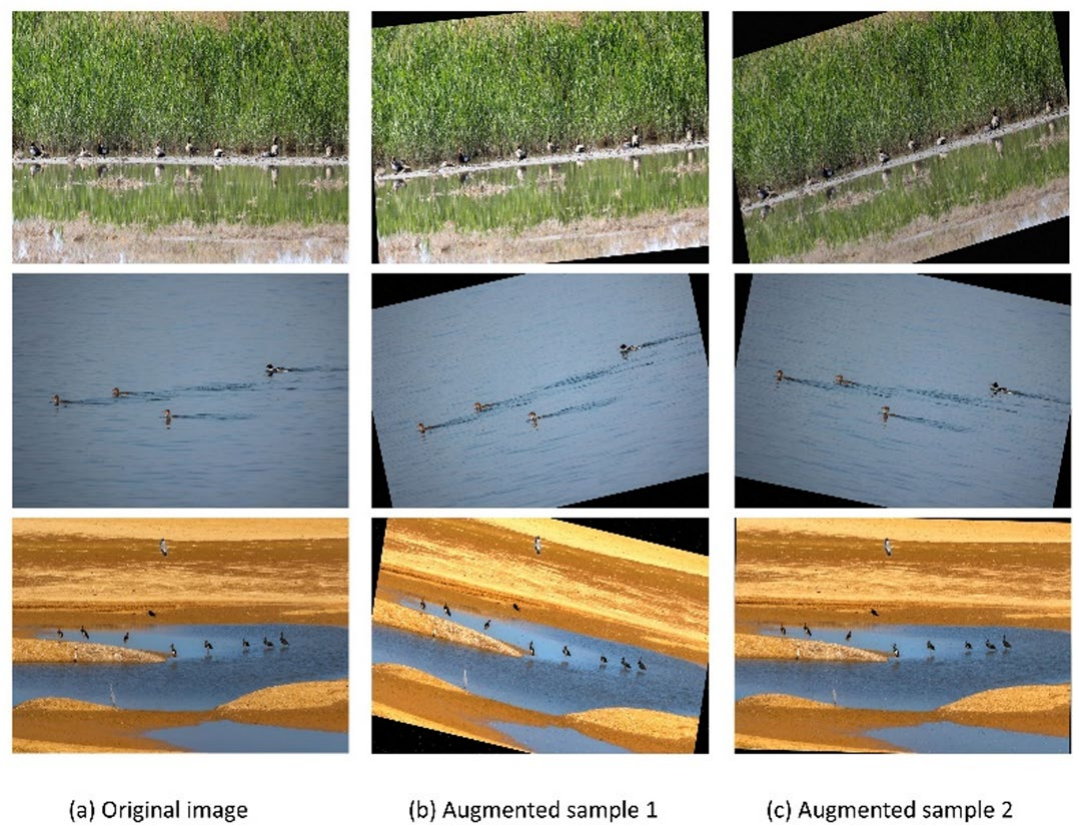
The samples of the offline data augmentation.

#### RLCB-YOLO model

**Fig. 4:**
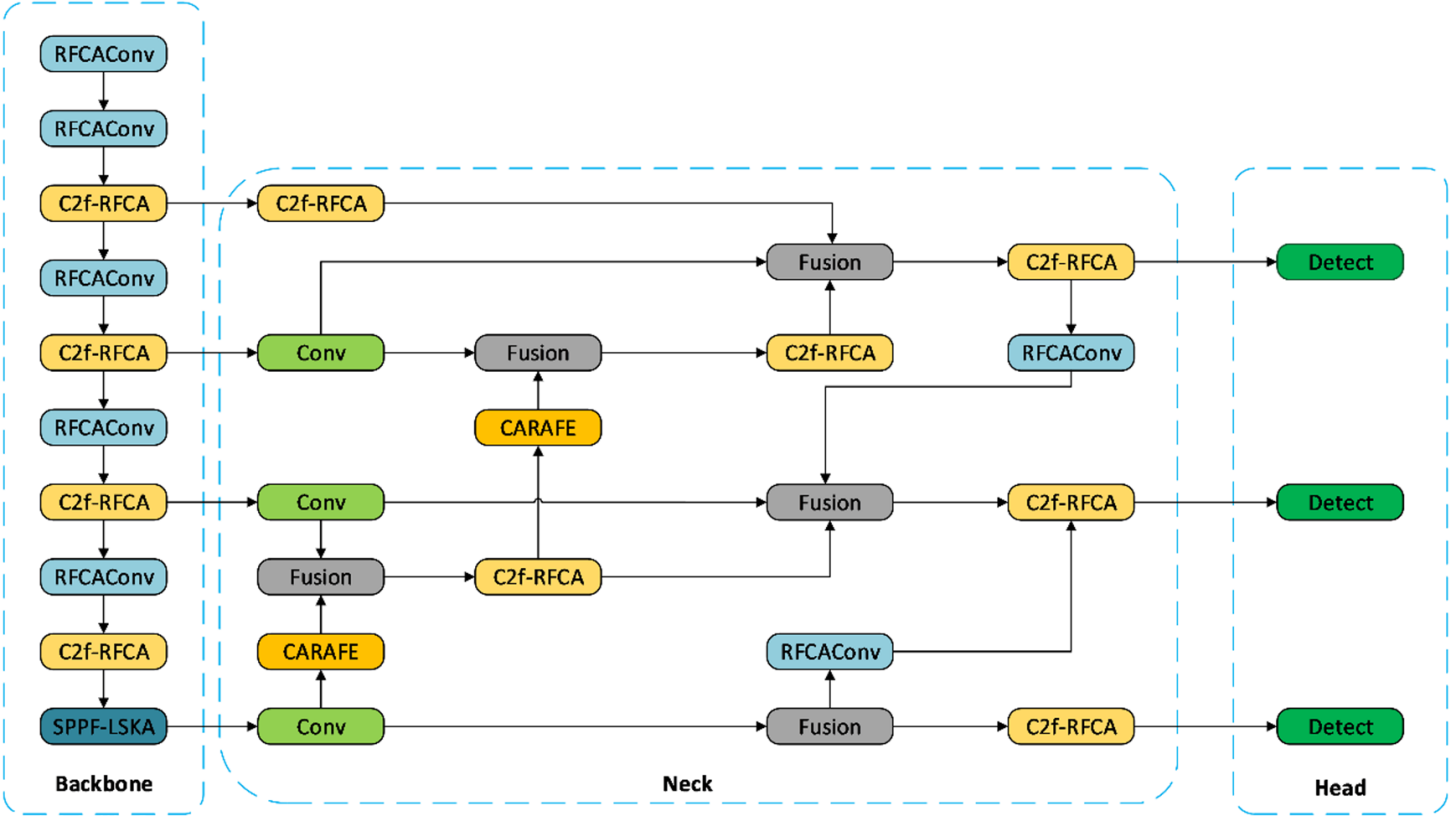
Structure of improved RLCB-YOLO network.

The YOLOv8n baseline provides the base for the proposed RLCB-YOLO, illustrated in Figs. 3, 4, to solve the difficult task of wetlands bird detection. To address challenges such as minute target scales, cluttered backgrounds, and significant reflection interference, systematic improvements were integrated into the backbone, context modeling, feature fusion, and up-sampling stages. Specifically, the backbone utilizes RFCAConv and C2f_RFCA to reconstruct the original Conv and C2f modules, thereby bolstering local fine-grained feature representation. In the high-level semantic stage, the SPPF-LSKA module is incorporated to expand the effective receptive field and enhance long-range contextual modeling. Within the Neck, a BiFPN-P2 structure is constructed to facilitate the transmission of high-resolution details during cross-scale fusion. The CARAFE module is introduced into the up-sampling branch to mitigate detail aliasing and structural information loss inherent in fixed interpolation. Collectively, these enhancements significantly improve the detection performance of small avian targets in challenging wetland environments.

### Model improvement

#### Receptive-field coordinate feature extraction module

In object detection tasks, attention mechanisms enhance the network’s ability to respond to key regions. Although Coordinate Attention (CA) introduces positional information through global pooling in horizontal and vertical directions, it essentially generates unified attention weights across the entire feature map. However, in complex wetland environments, birds often exhibit strong camouflage, as their feather colors are highly similar to backgrounds such as reeds and water grass. Traditional convolutional operators or CA mechanisms share the same parameter weights across different spatial locations, leading to homogenized local responses. This makes it difficult to capture the subtle texture differences between the targets and the background.

**Fig. 5:**
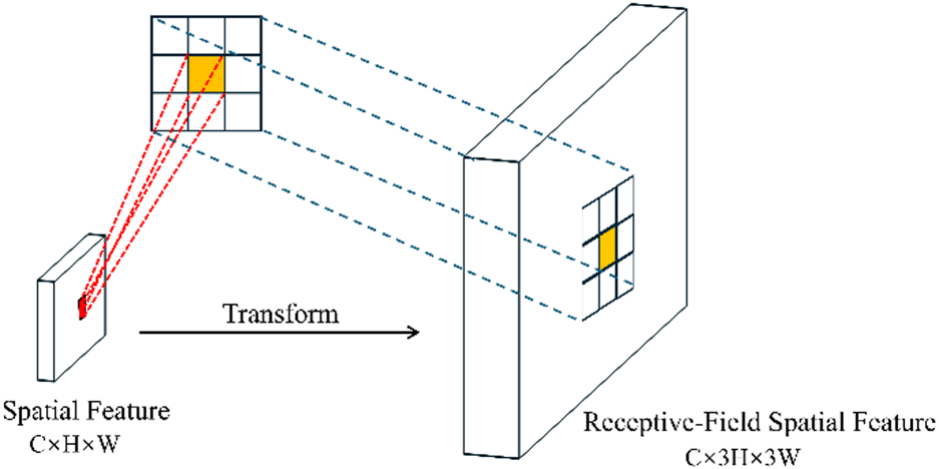
The spatial features are transformed to obtain the receptive-field spatial features.

Our research provides the RFCAConv module, which is based on the Receptive-Field Attention mechanism(RFA). The core idea of RFA is to shift the attention modeling domain from pixel space to receptive-field space^27^. It particularly produces position-adaptive attention weights for each sliding window, ensuring that the convolution is not using same weights across various spatial locations, which allows for different local responses. As illustrated in Fig. 5 (using a 3 × 3 kernel as an example), the original spatial features are expanded into the channel dimension via a sliding window and reorganized into structured receptive-field spatial features. Each 3 × 3 local region corresponds to an independent receptive field of the input feature map, allowing attention to be modeled independently at the window level.

**Fig. 6:**
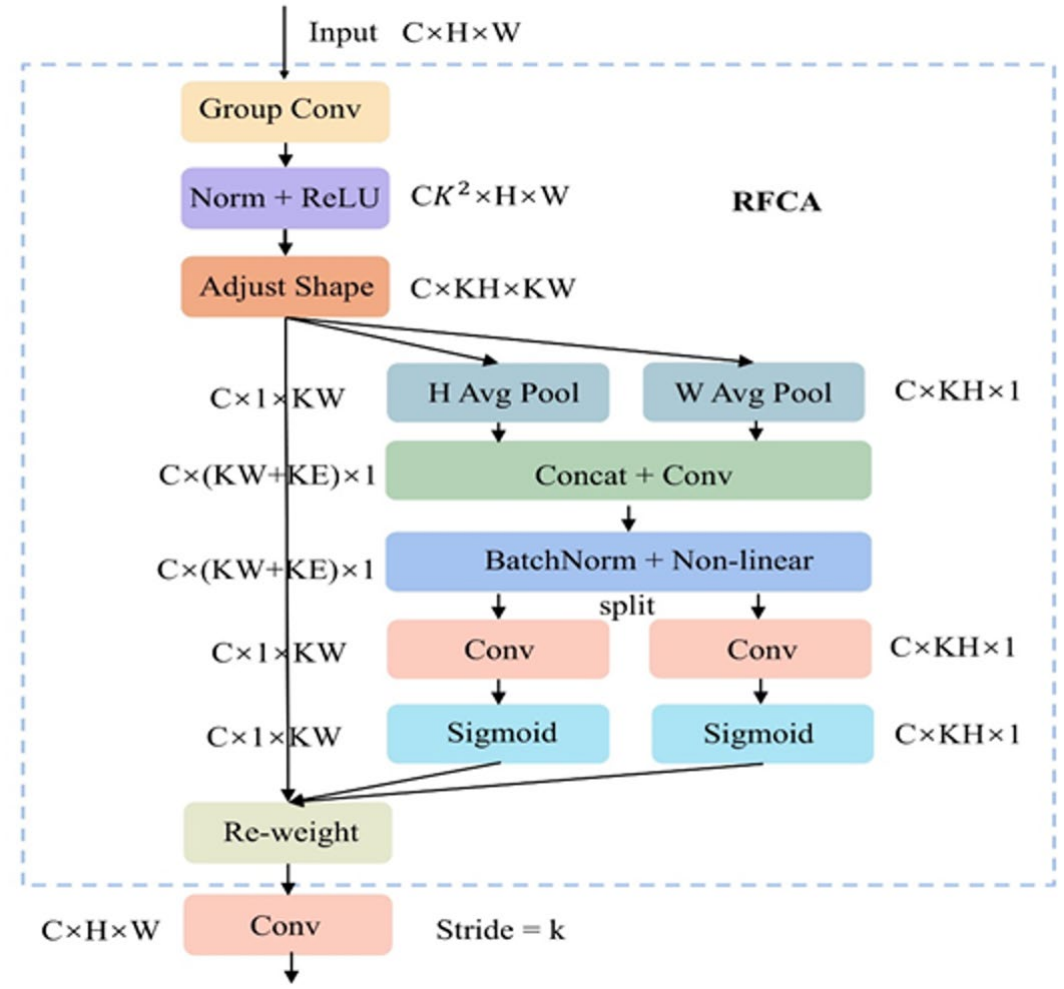
Structure of RFCAConv.

The architectural design of RFCAConv is illustrated in Fig. 6. The input feature maps first undergo group convolutions (Group Conv) to extract localized structural features within sliding windows. Following normalization and nonlinear activation, these features are reorganized into independent receptive-field units through an Adjust Shape operation. The RFCA module then builds two directionally encoded vectors for each receptive-field unit through average pooling in both horizontal and vertical directions. After being concatenated along the channel dimension and fused using batch normalization and activation, these vectors are divided into two branches. Each branch uses a convolution and a Sigmoid function to generate position-adaptive attention weights. These attention maps are applied to the corresponding receptive-field features via element-wise multiplication, achieving re-weighting at the sliding-window level. The re-weighted features then change back to the original size using a final convolution, ensuring that the output has the same resolution as the input.

**Fig. 7:**
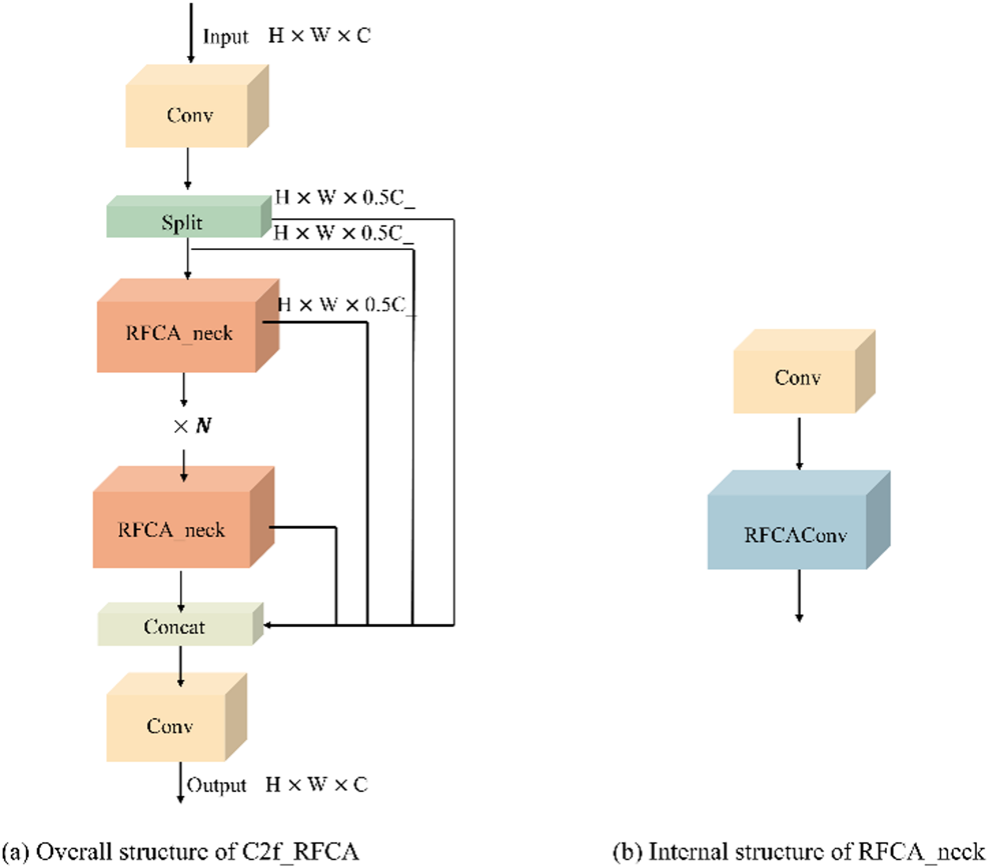
Structure of the proposed C2f_RFCA module. (**a**) Overall architecture of C2f_RFCA. (**b**) Internal structure of RFCA_neck.

By modeling attention within the receptive-field space, the RFCAConv module effectively mitigates the feature consistency issue caused by convolutional parameter sharing. The module incorporates directional encoding along horizontal and vertical axes, enhancing the network’s sensitivity to variations in spatial coordinates. This emphasizes the structural and border signals of small bird targets within complex environments, enhancing separability and discriminative stability between targets and backgrounds. Our research improved the original C2f module in YOLOv8 by introducing the RFCA_neck module, which upgrades the standard Bottleneck structure. In the RFCA_neck design, the original convolution layer is retained, while the subsequent convolution layers replaced by RFCAConv. Since the RFCA module already incorporates a residual connection for information fusion, additional shortcut connections are omitted to reduce computational redundancy. The resulting structure is illustrated in Fig. 7.

#### Improved SPPF-LSKA module

Due to environmental interferences such as water reflections, dense vegetation, frequent occlusion, and lighting fluctuations in wetland scenes, small bird targets often show low contrast and weak local details, making them easily confused with background textures. The model must expand its receptive field to incorporate contextual constraints while simultaneously suppressing false responses triggered by complex backgrounds. In order to solve this challenge, we built the SPPF-LSKA architecture by incorporating the Large Separable Kernel Attention (LSKA) mechanism into the YOLOv8 SPPF module. This modification can enhance the global perception and long-range dependency modeling of multi-scale features, effectively mitigating interference from complex backgrounds.

**Fig. 8:**
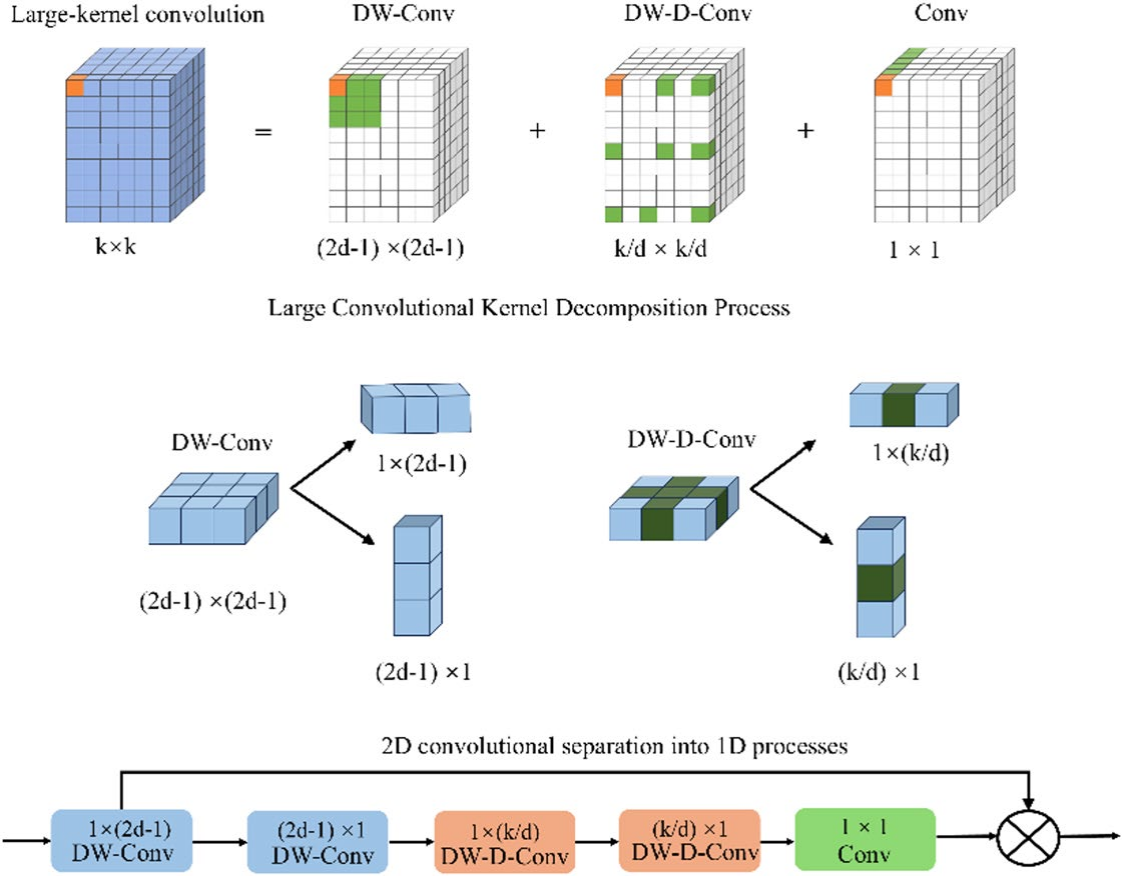
The LSKA attention structure.

LSKA is an evolution of Large Kernel Attention (LKA), integrating large-kernel receptive fields with separable convolutions. LSKA further decomposes the standard depth-wise convolution (DW-Conv) and depth-wise dilated convolution (DW-D-Conv) from LKA into 1D horizontal and vertical kernels, effectively balancing local details with global context^28^. As showed in Fig. 8, a K×K large-kernel convolution is equivalently represented by a cascaded sequence consisting of a (2d-1)×(2d-1) depth-wise convolution, a (K/d)×(K/d) depth-wise dilated convolution, and a 1 × 1channel-wise convolution. By dividing the 2D depth-wise kernels into consecutive 1D horizontal and vertical kernels, LSKA maintains an equivalent big receptive field and long-range modeling capacity while significantly reducing the computational burden usually linked to large-kernel attention.

**Fig. 9:**
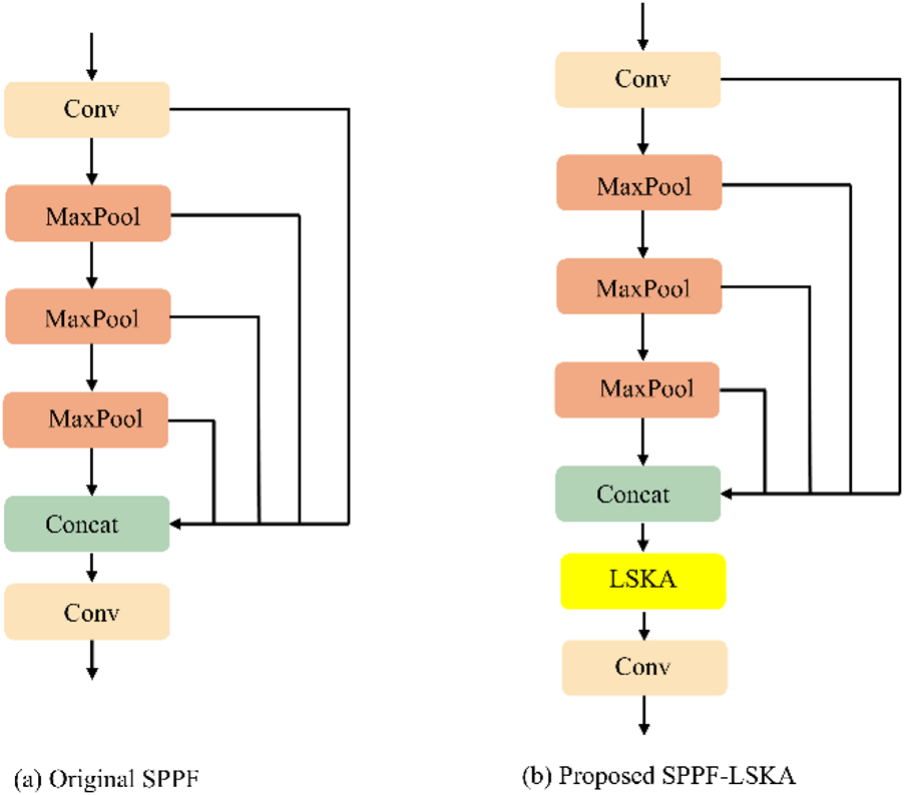
Comparison between the original SPPF and the proposed SPPF-LSKA.

Figure 9 illustrates the SPPF-LSKA architecture. High-level semantic features are extracted by SPPF-LSKA before multi-scale context branches are built using a series of serial max-pooling operations. These pooling outputs are then concatenated with the original branch along the channel dimension to form a fusion feature map. Our research added an LSKA module for adaptive recalibration after the concatenation stage, compared to the conventional SPPF, which only uses pooling and concatenation. By modeling spatial correlations across a larger receptive field, LSKA strengthens the response regions consistent with the structural features of birds while effectively suppressing interference from water reflections and complex vegetation textures. And a 1 × 1 convolution is applied for channel fusion, ensuring the output dimensions match the subsequent layers. Consequently, SPPF-LSKA enhances high-level context modeling with minimal computational overhead, providing a stable semantic prior for cross-scale fusion and improving detection performance in complex wetland environments.

#### The improved BiFPN-P2 structure

**Fig. 10:**
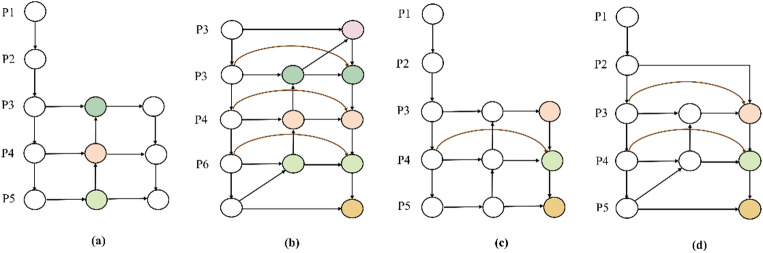
(**a**) The PAN structure. (**b**) The BiFPN structure. (**c**) The typical use of BiFPN in the YOLO. (**d**) The BiFPN-P2 structure.

In multi-scale feature fusion, the standard YOLOv8 Neck utilizes the Path Aggregation Network (PAN)^29^. In order to facilitate the transfer of low-level localization and edge details to high-level semantic features, PAN adds a bottom-up path aggregation to the top-down semantic propagation of FPN^30^. This dual-path flow significantly strengthens cross-scale representation capabilities, as illustrated in Fig. 10(a). BiFPN optimizes cross-scale information interaction by building a more compact bidirectional connection topology and simplifying single-input nodes that contribute little to the fusion in order to further enhance this process^31^. This structure enables high-level semantics and low-level details to be cyclically propagated and fused more efficiently. Moreover, the BiFPN structure supports stacking to achieve multiple rounds of bidirectional fusion, as shown in Fig. 10(b).

In conventional YOLO-based architectures, BiFPN is predominantly employed to enhance bidirectional fusion between mid- and high-level features, as illustrated in Fig. 10(c). However, for small bird targets in wetland environments, successive down-sampling operations significantly reduce the representation area in high-level feature maps. As a result, important information steadily decreases during the propagation process, including edges, contours, and weak textures. While relying primarily on the P3 level and above for feature fusion yields robust semantic representations, it often lacks the local structural details required to recover distant or minute objects. In contrast, the P2 layer offers higher spatial resolution and retains richer border and texture information, making it indispensable for the effective detection of small targets.

Based on the above analysis, our research introduce a P2 branch into BiFPN to construct the BiFPN-P2 structure, as illustrated in Fig. 10(d). Specifically, RFCAConv is first employed to recalibrate the P2 layer features, highlighting local regions and key textures relevant to avian targets while suppressing complex background interference. Strided convolution is then used to ensure down-sampling alignment, allowing for seamless incorporation into the bottom-up propagation path of BiFPN. The processed P2 features are then fused with mid- and high-level features, and the fused representation is further refined using C2f_RFCA to continuously inject low-level, fine-grained structural information during the cross-scale interaction process. Through these enhancements, BiFPN-P2 strengthens the transfer of high-resolution details to mid- and high-level semantic features. This improves the recognition and localization stability of small bird targets against complex wetland backgrounds and achieves efficient cross-scale feature fusion with controllable computational overhead.

#### Content-aware feature reorganization module

**Fig. 11:**
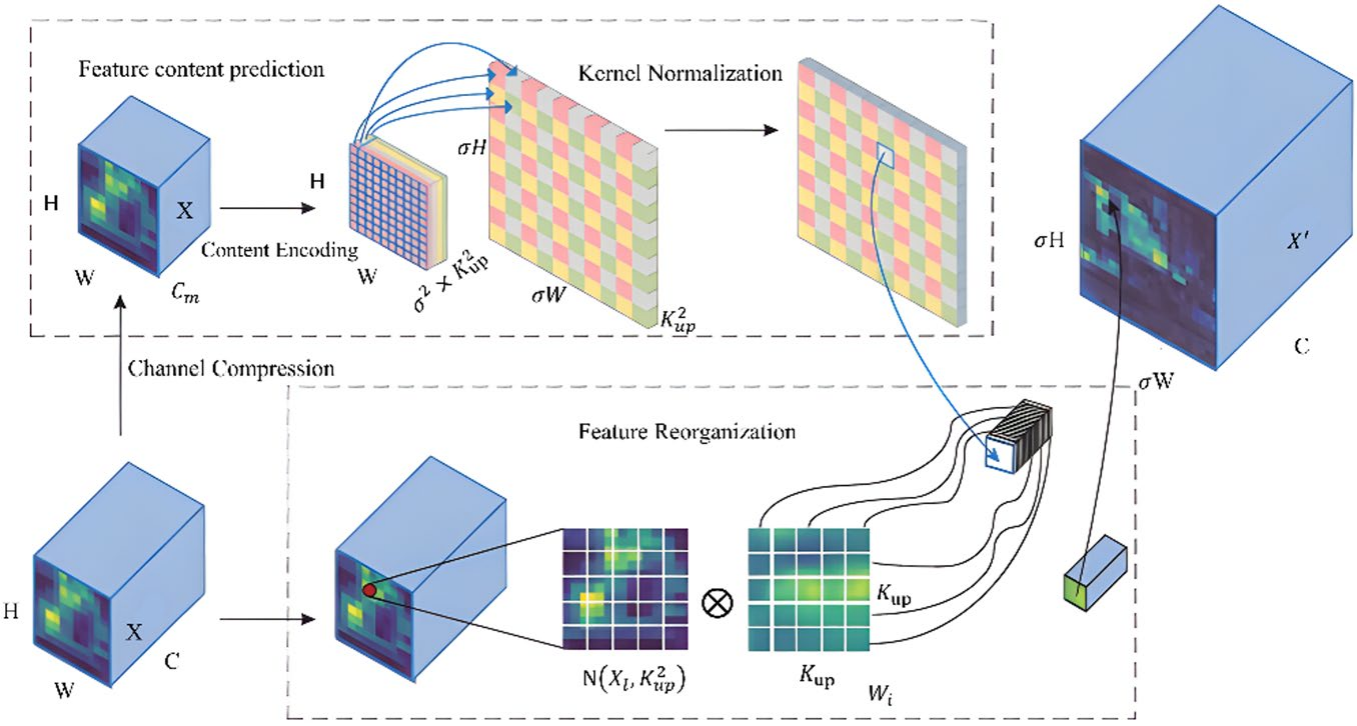
Module of content-aware feature reassembly.

Feature up-sampling is an essential process in feature fusion, particularly designed to restore high-resolution feature representations. Conventional techniques, like nearest-neighbor or bilinear interpolation, cannot adaptively reassemble features based on input content since they depend on fixed local geometric connections. In wetland environment, these methods often trigger feature aliasing due to water reflections, dense reeds, and complex backgrounds, leading to blurred boundaries and weakened local structures for small bird targets. In order to solve this problem, we replaced the standard structure in the up-sampling branch of the Neck with the Content-Aware ReAssembly of Features (CARAFE) module. CARAFE performs weighted reconstruction over a wide region and adaptively predicts reassembly kernels at each up-sampling position depending on surrounding features^32^. This approach combines a large receptive field with content adaptability. Compared to fixed interpolation, CARAFE is more effective at preserving discriminative information related to target boundaries and textures. It greatly improves the stability of feature recovery for small wetland birds by reducing background interference and information aliasing during feature alignment. The overall architecture is illustrated in Fig. 11.

The CARAFE module primarily comprises two components: a kernel prediction module and a feature reassembly module. Given an input feature map $\:\mathrm{X}\in\:{\mathrm{R}}^{\mathrm{C}\times\:\mathrm{H}\times\:\mathrm{W}}$, using $\:1\:\times\:1$ convolution is first applied to compress the channel dimension to$\:{\mathrm{C}}_{\mathrm{m}}$, yielding $\:{\mathrm{X}}_{\mathrm{c}}\in\:{\mathrm{R}}^{{\mathrm{C}}_{\mathrm{m}}\times\:\mathrm{H}\times\:\mathrm{W}}$to minimize computational overhead. Subsequently, the compressed features undergo content encoding to generate a reassembly kernel coefficient tensor of size $\:{{\upsigma\:}}^{2}{K}_{up}^{2}\times\:H\times\:W$. Next softmax normalization, the sum of the $\:{{\upsigma\:}}^{2}{\mathrm{K}}_{\mathrm{u}\mathrm{p}}^{2}$ weights at each spatial location equals one. This tensor is then rearranged into $\:{\upsigma\:}\mathrm{H}\times\:{\upsigma\:}\mathrm{W}\times\:{\mathrm{K}}_{\mathrm{u}\mathrm{p}}^{2}$. In the feature reassembly phase, for any target position $\:\mathrm{l}$ in the output feature map, CARAFE identifies a $\:{K}_{up}\times\:{K}_{up}$ neighborhood $\:\mathcal{N}\left(l\right)$ centered at its corresponding source position in the input map $\:\mathrm{X}$. The output feature vector $\:\mathrm{Y}\left(\mathrm{l}\right)$ is then computed as a weighted sum of the features within this neighborhood using the predicted reassembly kernel $\:{\mathrm{w}}_{\mathrm{l}}\left(\mathrm{j}\right)$:

1 $$\:Y\left(l\right)={\sum\:}_{j\in\:\mathcal{N}\left(l\right)}{w}_{l}\left(j\right)\hspace{0.17em}X\left(j\right)$$

$\:\mathrm{X}\left(\mathrm{j}\right)$ and $\:\mathrm{Y}\left(\mathrm{l}\right)$ represent the channel features at positions $\:\mathrm{j}$ and $\:\mathrm{l}$. All channels at the same spatial location share a unified set of up-sampling kernels. This operation produces an output feature map of size $\:{\upsigma\:}\mathrm{H}\:\times\:{\upsigma\:}\mathrm{W}\:\times\:\mathrm{C}$. In addition to keeping high-level semantic information during up-sampling, CARAFE effectively restores the detailed textures of bird targets against complex wetland backgrounds by using content-adaptive modeling and a large effective receptive field. This greatly increases the model’s sensitivity to small-scale bird objects.

## Experimental results and analysis

### Experimental equipment and environment

The experimental setup includes an I5-13490 F CPU and an NVIDIA GeForce RTX 4060 Ti GPU for hardware, and the software environment consisting of the 64-bit Windows 11 operating system, Python 3.8.18, PyTorch 2.1.1, and CUDA 12.1. YOLOv8n served as the baseline model for training. The detailed hyperparameters of the experimental environment are presented in Table 2.

**Table 2:** Training hyperparameter settings.

Hyperparameters	Value	Hyperparameters	Value
epochs	200	close_mosaic	10
patience	50	warmup_epochs	3.0
batch	8	lrf	0.01
images_size	640	lr0	0.01
workers	8	momentum	0.937
optimizer	SGD	weight_decay	0.0005

All models have an input image size of 640 × 640 and are trained using the same training parameters in the same experimental environment, each for 200 epochs. If the model shows no performance improvement over approximately 50 training epochs, the early stopping mechanism will be triggered to terminate the training. All YOLO models are trained on the dataset using the settings illustrated in Table 2.

### Evaluation metrics

To evaluate the performance of the improved model, this paper employs several metrics: precision (P), recall (R), mean average precision (mAP), frames per second (FPS), floating-point operations (FLOPs), and the number of trainable parameters (Params).

Precision (P) is the ratio of correctly predicted positive results to all predicted positive results. It is defined as shown in Eq. (2).

2 $$\:P=\frac{TP}{TP+FP}$$

Recall (R) is the proportion of actual positives correctly identified by the model. It is defined as shown in Eq. (3).

3 $$\:R=\frac{TP}{TP+FN}$$

Where TP denotes the number of correctly predicted samples, FP indicates the number of incorrectly predicted samples, and FN refers to the number of correct targets that were missed.

Mean average precision (mAP) considers both precision (P) and recall (R) across m classes, providing a comprehensive reflection of network performance. Mean detection accuracy includes two indicators: mAP@0.5 and mAP@0.5:0.95. By selecting different confidence thresholds, different PR curves can be obtained. It is defined as shown in Eq. (4).

4 $$\:mAP=\frac{1}{m}{\sum\:}_{i=1}^{m}{\int\:}_{0}^{1}P\left(R\right)dR$$

FPS measures the number of images the model can detect per second. In object detection tasks, when FPS is greater than 30, the model meets the requirements for real-time detection.

FLOPs (G) represent the number of floating-point operations required for a single inference, whereas Params (M) denote the total number of trainable parameters. These metrics reflect the computational complexity and architectural scale of the model. Lower values generally indicate a more lightweight model that is more suitable for deployment under resource-constrained conditions.

### Comparison of improved method effects

#### Comparative experiment of different convolutions

To verify the performance of different variant convolutions in YOLOv8 and confirm the efficacy of RFCAConv in complex wetland bird detection tasks, we tested various convolution modules, including RFAConv, RFCAConv and RFCBAMConv^27^. The experimental results are illustrated in Table 3.

**Table 3:** Comparison of different convolutional modules.

Model	Precision (%)	Recall(%)	mAP@0.5(%)	mAP@0.5:0.95 (%)	Params (M)	FLOPs (G)
RFAConv	87.1	73.2	80.0	46.4	3.13	8.8
RFCBAMConv	88.2	72.8	80.4	46.7	3.16	8.9
RFCAConv	**86.9**	**74.1**	**81.0**	**47.3**	**3.13**	**8.8**

According to experimental results, RFCAConv keeps a computational cost and parameter count that are essentially on par with other variations while achieving optimal comprehensive performance. RFCAConv improves RFAConv by 1.0 and 0.9% points, achieving a mAP@0.5 of 81.0% and a mAP@0.5:0.95 of 47.3%. It also surpasses the RFCBAMConv module, which incorporates the CBAM attention mechanism. Additionally, RFCAConv achieves the highest recall rate of 74.1% out of the three modules, confirming its remarkable ability to detect small targets against complex backgrounds. These findings indicate that by Coordinate Attention into the receptive field space, RFCAConv accurately reflects spatial features in both horizontal and vertical directions, then significantly enhancing bird detection performance in wetland environments.

#### Comparative experiment of different attention mechanisms improving SPPF

To evaluate the effects of combining different attention mechanisms with the SPPF module, this study designed a series of comparative experiments. Specifically, SPPF modules integrated with SE^33^, CBAM^34^, CA, LSK, and LSKA attention mechanisms were compared. The experimental results are illustrated in Table 4.

**Table 4:** Improvement of the SPPF module using different attention mechanisms.

Model	Precision (%)	Recall(%)	mAP@0.5(%)	mAP@0.5:0.95 (%)	Params (M)	FLOPs (G)
SPPF-SE	86.9	71.2	78.5	45.9	3.03	8.1
SPPF-CA	87.5	71.8	79.1	46.1	3.03	8.1
SPPF-CBAM	87.1	72.0	79.5	46.1	3.26	8.1
SPPF-LSK	88.2	71.8	79.7	46.2	3.56	8.8
**SPPF-LSKA**	**88.5**	**71.5**	**79.8**	**46.4**	**3.27**	**8.3**

As the results show, SPPF-LSKA performs best in model accuracy, with mAP@0.5 and mAP@0.5:0.95 reaching 79.8% and 46.4%, beyond the SPPF-SE, SPPF-CA, and SPPF-CBAM. This result confirms that introducing the LSKA mechanism into the SPPF significantly enhances the ability to recognize small bird targets in complex wetland environments. Although the FLOPs of SPPF-LSKA are slightly higher than those of SE, CBAM, and CA, the significant performance improvement justifies the additional computational cost. This indicates that the introduction of the LSKA mechanism achieves an effective balance between excellent detection accuracy and reasonable computational burden.

#### Comparison of different feature fusion strategies within the BiFPN

Cross-scale information interaction is significantly impacted by fusion strategies during BiFPN-P2 feature fusion. Our research compared Weight, Adaptive, and Concat strategies against the default BiFPN fusion with the same structural and training settings in order to evaluate their effect on performance. The experimental results are illustrated in Table 5.

**Table 5:** Performance of the BiFPN-P2 structure using different fusion strategies.

Model	Precision (%)	Recall(%)	mAP@0.5(%)	mAP@0.5:0.95 (%)	Params (M)	FLOPs (G)
Weight	88.0	70.7	79.4	46.4	2.04	7.5
Adaptive	85.6	71.8	79.1	46.0	2.04	7.5
Concat	87.3	70.6	78.7	45.1	2.01	7.2
**BiFPN**	**86.5**	**70.9**	**79.3**	**46.4**	**1.99**	**7.1**

The Weight method optimizes fusion by assigning learnable weights to feature map s from different layers. The Adaptive method dynamically adjusts the fusion strategy based on inherent feature properties. The Concat method joins feature maps from various scales directly along the channel dimension. The BiFPN approach uses a bidirectional fusion mechanism for comprehensive feature aggregation. Experimental results indicate that although the Weight-based method slightly outperforms the default BiFPN in mAP@0.5 by 0.1% point, it entails a higher computational overhead, with FLOPs (G) increasing from 7.1 to 7.5. Considering the trade-off between detection performance and computational cost, the default BiFPN fusion strategy was adopted within the BiFPN-P2 structure in the final model.

#### Ablation experiment

Using YOLOv8n as the baseline, ablation experiments were performed to validate the improvements of RFCAConv, SPPF-LSKA, BiFPN-P2, and CARAFE for small bird targets detection in wetland environments. All experiments were executed under consistent training protocols and a standardized dataset. The experimental results are illustrated in Table 6.

**Table 6:** Results of ablation experiments.

Method	Precision (%)	Recall(%)	mAP@0.5 (%)	mAP@0.5:0.95 (%)		Params (M)	FLOPs (G)
YOLOv8n	87.3	71.1	78.5	45.7		3.01	8.2
+RFCAConv	86.9	74.1	81.0	47.3		3.13	8.8
+SPPF-LSKA	88.5	71.5	79.8	46.4		3.27	8.3
+BiFPN-P2	86.5	70.9	79.3	46.4		1.99	7.1
+CARAFE	88.1	72.2	80.2	45.9		3.14	8.4
+RFCAConv+SPPF-LSKA	89.4	72.4	81.5	47.4		3.41	9.1
+RFCAConv+BiFPN-P2	89.0	73.0	81.2	47.9		2.10	7.9
+RFCAConv+BiFPN-P2 + CARAFE	89.3	73.3	81.4	47.7		2.48	8.2
**+RFCAConv+ SPPF-LSKA+BiFPN-P2 + CARAFE**	**89.0**	**75.0**	**82.1**	**48.6**		**2.50**	**8.4**

As indicated by the results in the table, the individual integration of each module enhances performance to different degrees. The addition of the RFCAConv module increases the recall rate from 71.1% to 74.1%, a significant rise in parameters, and the mAP@0.5 from 78.5% to 81.0%, a gain of 2.5%. This indicates that differential attention within the receptive field space effectively bolsters the modeling of fine-grained textures and local structures for small targets. The improved SPPF-LSKA module boosts the mAP@0.5 to 79.8% and precision from 87.3% to 88.5%, proving that long-range dependency modeling strengthens target region responses in complex backgrounds. The BiFPN-P2 structure simultaneously increases the mAP@0.5 to 79.3% while lowering the parameter count from 3.01 M to 1.99 M, showing the effectiveness of the high-resolution feature path for achieving a lightweight yet accurate architecture. The CARAFE module increases the mAP@0.5 to 80.2% and the recall rate to 72.2%, indicating that the content-adaptive reassembly mechanism is crucial for preserving structural information of dense small targets during up-sampling. Experiments involving multiple module combinations further validate the synergistic gains of the proposed enhancements. The integration of RFCAConv with SPPF-LSKA increased the mAP@0.5 to 81.5%, while its combination with BiFPN-P2 reached 81.2%. Further incorporating CARAFE yielded an mAP@0.5 of 81.4% and an mAP@0.5:0.95 of 47.7%, demonstrating a strong complementary nature among feature enhancement, long-range dependency modeling, and content-aware up-sampling. The mAP@0.5 and mAP@0.5:0.95 reached 82.1% and 48.6% in the final RLCB-YOLO model, which combines all four modules. Compared to the baseline YOLOv8n, the proposed model achieves a substantial improvement in detection accuracy while maintaining nearly identical FLOPs and reducing the parameter count from 3.01 M to 2.50 M. The results illustrate that the proposed module combination achieves an optimal balance between detection performance and computational efficiency.

### Hyperparameter sensitivity analysis

#### Sensitivity analysis of input image size

To evaluate the performance robustness of the proposed model over different input scales and to verify whether the detection improvements are resolution-independent. Comparative experiments were performed between the baseline YOLOv8n and the proposed RLCB-YOLO. While keeping all other training parameters constant, our research evaluated three input image sizes: 512 × 512, 640 × 640, and 768 × 768. Table 7 shows the experimental results.

**Table 7:** Sensitivity analysis under different input image sizes.

Method	Input Size	Precision(%)	Recall(%)	mAP@0.5(%)	mAP@0.5:0.95(%)
YOLOv8n	512 × 512	86.6	70.2	77.0	43.8
YOLOv8n	640 × 640	87.3	71.1	78.5	45.7
YOLOv8n	768 × 768	88.2	71.9	79.3	47.4
RLCB-YOLO	512 × 512	88.2	73.1	80.5	47.2
RLCB-YOLO	640 × 640	89.0	75.0	82.1	48.6
RLCB-YOLO	768 × 768	88.6	74.6	81.5	49.1

Table 7 shows that variations in input image size significantly influence the detection performance of both models. As the input resolution scales from 512 × 512 to 768 × 768, the Precision, Recall, and mAP metrics for YOLOv8n show a general upward trend. This indicates that higher resolutions help mitigate the information compression of small-scale bird targets in complex backgrounds. In contrast, RLCB-YOLO achieves its optimal comprehensive performance at a resolution of 640 × 640, with Precision, Recall, and mAP@0.5 reaching 89.0%, 75.0%, and 82.1%, respectively. When the input size is further increased to 768 × 768, the mAP@0.5:0.95 shows a marginal improvement from 48.6% to 49.1%, while other metrics slightly decline. This indicates that although larger input scales provide some gains under stricter evaluation criteria, they do not further enhance overall comprehensive performance. Overall, RLCB-YOLO maintains a performance advantage over YOLOv8n across all three tested resolutions, indicating that the proposed model remains stable within the evaluated input-size range. Therefore, 640 × 640 is selected as the default input size in this study, considering both deployment practicality and detection performance.

#### Sensitivity analysis of learning rate

To evaluate the training stability of the proposed model, our research conducted a sensitivity analysis centered on the default learning rate setting. Three initial learning rates—0.005, 0.01, and 0.02—were chosen to compare the performance of YOLOv8n with RLCB-YOLO while keeping all other training parameters constant. The experimental results are shown in Table 8. This experiment aims to determine whether the performance improvements of our model are dependent on a specific learning rate configuration.

**Table 8:** Sensitivity analysis under different initial learning rates.

Method	Input Size	Precision(%)	Recall(%)	mAP@0.5(%)	mAP@0.5:0.95(%)
YOLOv8n	0.005	88.5	70.0	78.1	44.9
YOLOv8n	0.010	87.3	71.1	78.5	45.7
YOLOv8n	0.020	88.3	71.0	78.8	46.5
RLCB-YOLO	0.005	88.2	73.8	80.8	47.5
RLCB-YOLO	0.010	89.0	75.0	82.1	48.6
RLCB-YOLO	0.020	88.9	74.4	81.7	49.0

For YOLOv8n, its recall peaks at lr0 = 0.01, and both mAP@0.5 and mAP@0.5:0.95 show a slight rising trend as the learning rate rises from 0.005 to 0.02. RLCB-YOLO achieves its optimal comprehensive performance at lr0 = 0.01, with Precision, Recall, and mAP@0.5 reaching 89.0%, 75.0%, and 82.1%, respectively. Although the mAP@0.5:0.95 marginally improves to 49.0% when the learning rate is further increased to 0.02, other indicators show only minor fluctuations. RLCB-YOLO constantly maintains higher Recall, mAP@0.5, and mAP@0.5:0.95 than YOLOv8n, despite the differences across various learning rate settings. This shows significant stability through a suitable optimization range, illustrating that the performance advantages of RLCB-YOLO are independent of a particular learning rate setting. As a result, the default initial learning rate for the experiments in this research is set at 0.01.

#### Comparative experiment

**Fig. 13:**
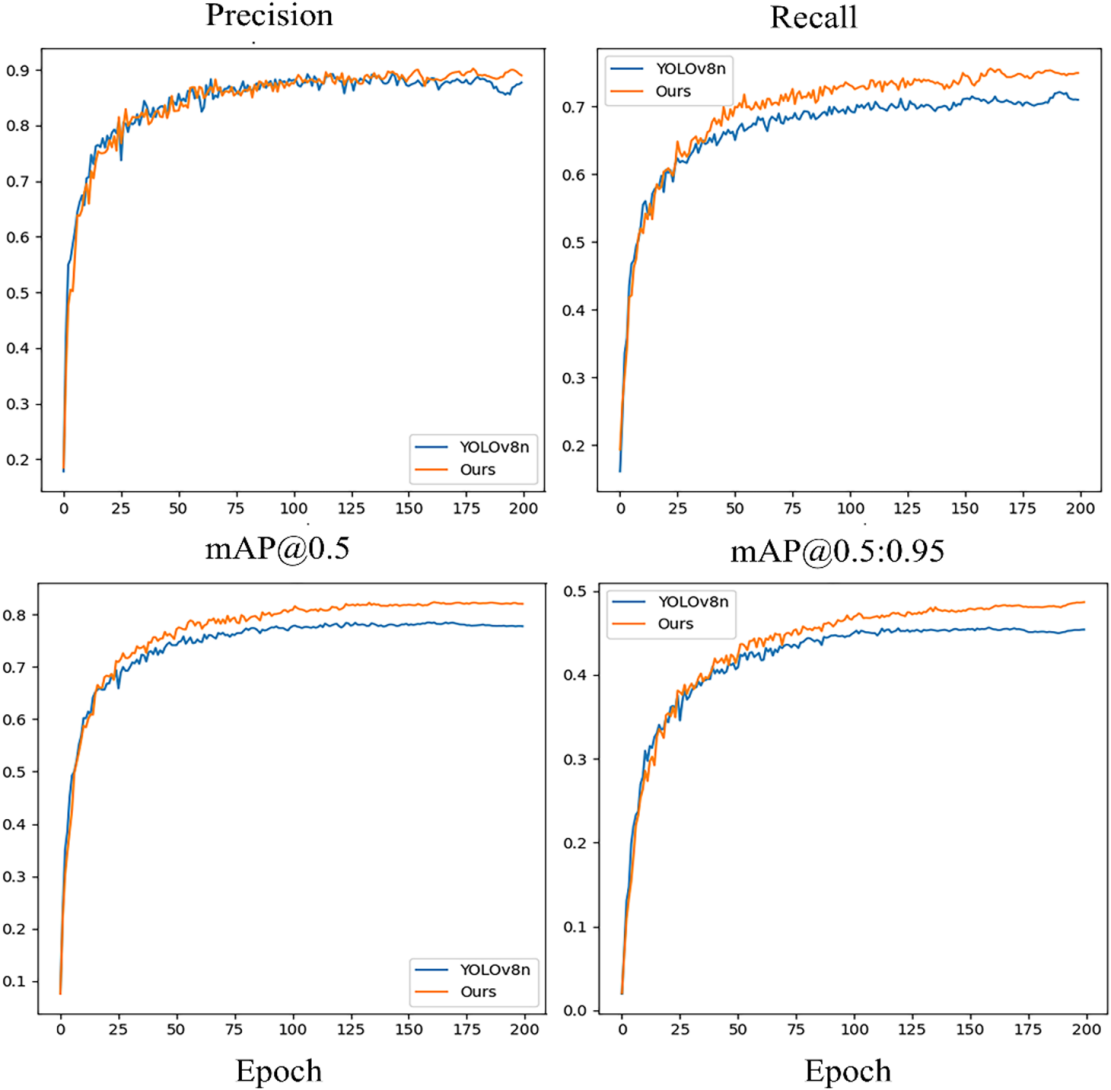
YOLOv8n and RLCB-YOLO performance comparison.

Adopting the setup described in the ‘Experimental Equipment and Environment’ section, we performed comparative training for RLCB-YOLO and YOLOv8n, focusing on the convergence of Precision, Recall, mAP@0.5, and mAP@0.5:0.95. The training was limited to 200 epochs, incorporating an early stopping mechanism with a patience of 50 epochs for mAP@0.5 improvement. Figure 13 displays these comparison results.

Figure 13 shows that while both models converge similarly in the early phase, RLCB-YOLO demonstrates a significant edge after 50 epochs. Significantly, its Recall curve demonstrates enhanced stability and superior accuracy as training progresses, emphasizing its robustness to detect small targets against cluttered backgrounds. For mAP@0.5 and mAP@0.5:0.95, RLCB-YOLO consistently leads the baseline from the mid-stage, achieving faster convergence and superior final stability. These results corroborate the ablation experiments and confirm the effectiveness of RLCB-YOLO in enhancing detection performance for challenging wetland environments.

#### Comparison with mainstream detection algorithms

Our research conducted a comparative analysis with other models in order to further evaluate RLCB-YOLO’s overall performance. These include Faster R-CNN, several YOLO variants, and RT-DETR. All experiments were performed on our specialized wetland bird dataset under a unified training protocol. The detailed performance metrics for each model are summarized in Table 9.

**Table 9:** Comparison results of different networks.

Method	Precision (%)	Recall(%)	mAP@0.5 (%)	mAP@0.5:0.95 (%)		Params (M)	FLOPs (G)	FPS
Faster R-CNN	81.2	63.5	71.2	41.3		41.39	208.1	32
YOLOv5n	86.9	70.1	77.8	45.1		2.53	7.8	218
YOLOv7-tiny	87.0	70.5	78.1	45.3		6.01	13.0	153
YOLOv8n	87.3	71.1	78.5	45.7		3.01	8.1	216
YOLOv8s	89.6	73.1	81.3	48.3		11.13	28.4	172
YOLOv11n	87.7	70.4	78.6	45.7		2.58	6.3	256
Hyper-YOLO	90.0	70.5	78.8	46.3		3.62	9.5	147
RT-DETR	89.2	75.3	81.9	48.5		20.09	56.9	110
**RLCB-YOLO**	**89.0**	**75.0**	**82.1**	**48.6**		**2.50**	**8.4**	**101**

As indicated by the results, RLCB-YOLO achieves an mAP@0.5 of 82.1% and an mAP@0.5:0.95 of 48.6%, representing improvements of 3.6 and 2.9% points over the baseline YOLOv8n. RLCB-YOLO increases accuracy while reducing the parameter count to 2.50 M, which represents a decrease of approximately 17%, thus indicating an improvement in detection precision and architectural efficiency. While the FPS decreased slightly, the proposed model still sustains high real-time detection performance at 101 FPS. RLCB-YOLO uses only 22.5% of its parameters and 29.6% of its computational load to achieve better accuracy than the larger YOLOv8s. RLCB-YOLO’s superior suitability for domain-specific tasks like wetland bird detection over general-purpose large-scale models is validated by the fact that it maintains comparable mAP while requiring only 14.8% of the FLOPs, even when compared to the Transformer-based RT-DETR. From a deployment viewpoint, Faster R-CNN’s large parameter scale and limited precision (mAP@0.5 of 71.2%) fall short of wetland monitoring’s real-time demands. As a result, RLCB-YOLO shows significant engineering potential and achieves an ideal balance between precision, model scale, and inference complexity.

#### Visualization analysis

To visually demonstrate the performance improvement of the improved model in detecting small bird targets in complex wetland environments, this study selected multiple representative scene images from the collected dataset. Under identical experimental conditions, we conducted a visual comparison between the original YOLOv8n model and the improved RLCB-YOLO model to present the effect differences brought by these improvements.

**Fig. 14:**
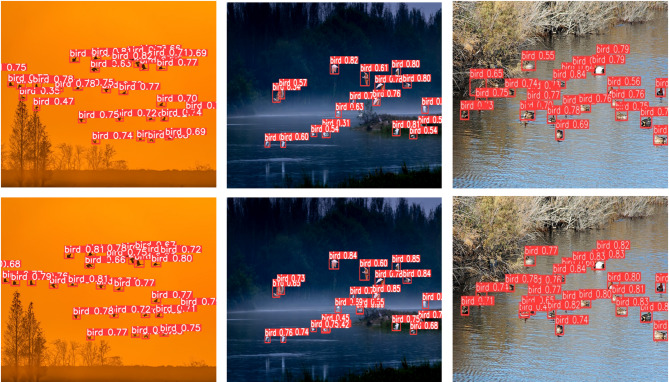
Comparison of dense target detection results. The first row shows the YOLOv8n detection results, and the second row shows the RLCB-YOLO detection results.

Figure 14 illustrates the performance of detecting dense bird targets in various scenarios. The original YOLOv8n model tends to exhibit false positives and missed detections in complex natural backgrounds, such as bird flocks under sunset illumination and birds on the water surface. In contrast, the RLCB-YOLO model demonstrates higher accuracy in detecting bird targets, regardless of the sunset lighting or water surface conditions. The improved model enhances the detection accuracy of bird targets, proving its superior performance in scenarios involving dense bird target detection.

**Fig. 15:**
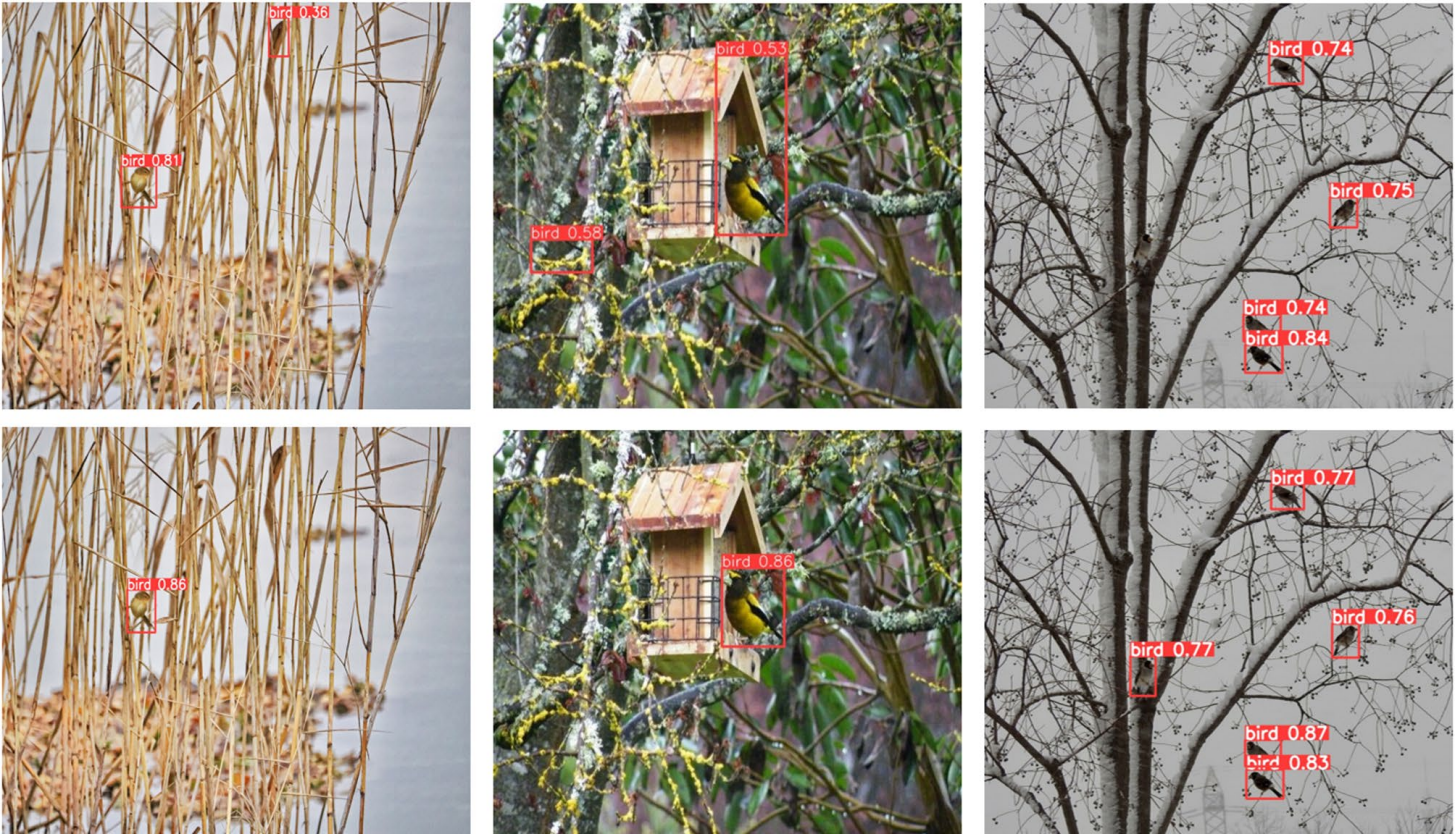
Comparison of detection results under environmental interference. The first row shows the YOLOv8n detection results, and the second row shows the RLCB-YOLO detection results.

Figure 15 illustrates that the original YOLOv8n model struggled to accurately identify bird targets when their colors were similar to the surrounding environment or the background was complex. The improved RLCB-YOLO model successfully identified and distinguished these targets, demonstrating its high recognition accuracy even with environmental interference.

**Fig. 16:**
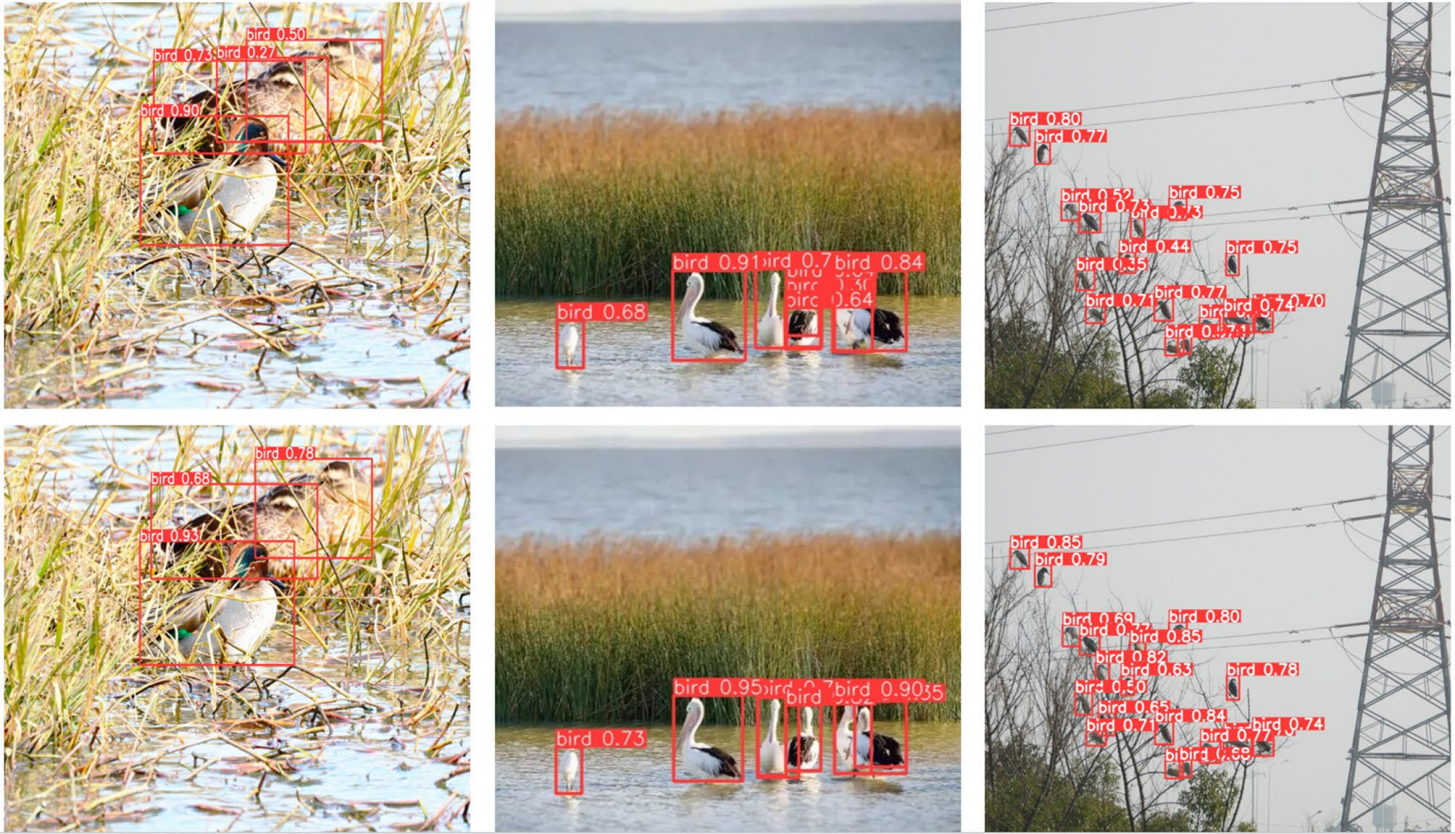
Comparison of detection results with occlusion overlap. The first row shows the YOLOv8n detection results, and the second row shows the RLCB-YOLO detection results.

Finally, Fig. 16 illustrates that under complex conditions of foliage occlusion and bird overlap, the RLCB-YOLO model accurately identifies targets, effectively avoiding common false positives and missed detections. Overall, the improved model has significantly improved over the original YOLOv8n model in detecting small bird targets in complex wetland environments.

## Discussion

In this study, we propose a deep learning-based object detection model for detecting small bird targets in complex wetland environments. Birds play a crucial role in wetland ecosystems. By monitoring the number of wetland birds, researchers can estimate their population size, distribution, and dynamic trends, thereby assessing the overall health of wetlands and formulating targeted bird conservation plans.

Traditional bird monitoring methods typically rely on manual field surveys, which are limited in complex and diverse wetland environments. Birds in wetland environments often inhabit areas that are difficult to access, such as shrubbery and swamp surfaces, limiting the efficiency and scope of manual detection. Although some researchers use drone aerial photography to cover a wide range, these devices require a professional operation^35^. Drones are also limited by flight time and distance, and their noise may disturb bird habitats^36^. While radar technology can provide rough information on flock size and migration direction^37,38^, it cannot accurately detect small targets in complex environments.

To accurately and efficiently detect birds in complex wetland environments, this study developed the RLCB-YOLO model based on YOLOv8n. This model significantly enhances the accuracy and efficiency of detecting small bird targets, effectively overcoming the limitations of existing methods in handling small targets and complex scenarios. Furthermore, the methods proposed in this study are not only applicable to wetland environments but can also be extended to other monitoring scenarios, such as tracking small animals in urban settings or monitoring wildlife in forests, demonstrating the broad potential of deep learning technology in ecological monitoring and biodiversity conservation.

Although this study has made significant progress in detecting small bird targets in wetlands, we still face certain limitations. First, current work focuses primarily on detecting a single type of bird. In the future, we plan to expand the model’s functionality to include the detection and identification of multiple bird species, enabling a more comprehensive study of wetland bird population changes. Secondly, the current dataset is relatively small. When faced with extremely complex backgrounds such as dynamic water reflections or dense vegetation, the model’s recognition accuracy and stability require further enhancement. To address these issues, we plan to collect a more diverse dataset and enhance it by using GANs^39^ to generate training images, thereby improving the model’s adaptability and performance under various environmental conditions. Additionally, we will explore applying the model to other types of small target monitoring, such as tracking small animals in urban environments or wildlife in forests, to test the model’s versatility and practicality. Through these measures, we aim to apply artificial intelligence technology more broadly in wildlife monitoring and environmental conservation, thereby contributing to ecological protection and biodiversity conservation.

The initial aim of this study was to develop an efficient and easily deployable bird detection method. To adapt to resource-limited environments, we plan to integrate the model with edge computing technology and further enhance the model to improve detection speed and overall performance^40,41^. This integration will enable the model to process and analyze data in real-time, expanding its application potential in field monitoring.

## Conclusions

To improve the accuracy and efficiency of detecting small bird targets in complex wetland environments, this study constructed a dataset of small bird targets in complex wetland environment and developed the RLCB-YOLO object detection model. The RFCAConv module is employed to reconstruct critical convolutional layers in the backbone, enhancing the discriminative power of local fine-grained features. Furthermore, the SPPF-LSKA module expands the effective receptive field and strengthens long-range contextual modeling, thereby mitigating interference from cluttered background textures and water reflections. The BiFPN-P2 structure helps the continuous injection of high-resolution details into the cross-scale fusion process, improving the alignment between small target features and high-level semantics. Additionally, in order to rebuild features in a content-aware manner and reduce detail confusing and structural information loss that are inherent in fixed interpolation, the CARAFE module is added during the up-sampling stage.

On the self-built small-scale wetland bird dataset, experimental results show that RLCB-YOLO achieves significant efficiency improvements. At 89.0%, 75.0%, 82.1%, and 48.6%, Precision, Recall, mAP@0.5, and mAP@0.5:0.95 bettered the baseline YOLOv8n by 1.7%, 3.9%, 3.6%, and 2.9%. The parameter count was reduced from 3.01 M to 2.50 M, ensuring that the computational footprint and model scale remain strictly within the lightweight category while delivering superior detection accuracy. Visualizations illustrate the model’s exceptional performance in detecting bird targets in complex wetland environments.

In conclusion, the results of this study demonstrate the potential application of deep learning technology in ecological research and provide technical support for wetland and bird conservation. In the future, we will continue to optimize the model’s performance and extend its application to various scenarios, thereby contributing to ecological monitoring and biodiversity conservation.

## Data Availability

The data and code used during the current study are available from the corresponding author upon reasonable request.

## References

[1] Dawson, T. P., Berry, P. M. & Kampa, E. Climate change impacts on freshwater wetland habitats. *J. Nat. Conserv.* **11**, 25–30. 10.1078/1617-1381-00031 (2003).

[2] Day, J. W. et al. Consequences of climate change on the ecogeomorphology of coastal wetlands. *Estuaries coasts*. **31**, 477–491. 10.1007/s12237-008-9047-6 (2008).

[3] Paracuellos, M. & Tellería, J. L. Factors affecting the distribution of a waterbird community: the role of habitat configuration and bird abundance. *Waterbirds* **27**, 446–453. 10.1675/1524-4695 (2004).

[4] Weller, M. W. *Wetland birds: habitat resources and conservation implications* (Cambridge University Press, 1999).

[5] Gaston, K. J. et al. Population abundance and ecosystem service provision: the case of birds. *BioScience* 68, 264–272. 10.1093/biosci/biy005 (2018). 10.1093/biosci/biy005 PMC5905662 29686433

[6] Gregory, R. D., Gibbons, D. W. & Donald, P. F. *Bird census and survey techniques* (2004).

[7] Du, N., Fathollahi-Fard, A. M. & Wong, K. Y. Wildlife resource conservation and utilization for achieving sustainable development in China: main barriers and problem identification. *Environ. Sci. Pollut. Res.* 1–20. 10.1007/s11356-023-26982-7 (2023). 10.1007/s11356-023-26982-7 PMC10122203 37086322

[8] Bakó, G., Tolnai, M. & Takács, Á. Introduction and testing of a monitoring and colony-mapping method for waterbird populations that uses high-speed and ultra-detailed aerial remote sensing. *Sensors* **14**, 12828–12846. 10.3390/s140712828 (2014). 25046012 10.3390/s140712828 PMC4168513

[9] Lomnicky, G. A., Herlihy, A. T. & Kaufmann, P. R. Quantifying the extent of human disturbance activities and anthropogenic stressors in wetlands across the conterminous United States: results from the National Wetland Condition Assessment. *Environ. Monit. Assess.* **191**, 324. 10.1007/s10661-019-7314-6 (2019). 31222443 10.1007/s10661-019-7314-6 PMC6586716

[10] Zhang, C. & Lu, Y. Study on artificial intelligence: The state of the art and future prospects. *J. Industrial Inform. Integr.* **23**, 100224. 10.1016/j.jii.2021.100224 (2021).

[11] Pan, Y. Heading toward artificial intelligence 2.0. *Engineering* 409–413. 10.1016/J.ENG.2016.04.018 (2016).

[12] Yousif, H., Yuan, J., Kays, R. & He, Z. Animal Scanner: Software for classifying humans, animals, and empty frames in camera trap images. *Ecol. Evol.* **9**, 1578–1589. 10.1002/ece3.4747 (2019). 30847057 10.1002/ece3.4747 PMC6392355

[13] Yang, L. et al. Computer vision models in intelligent aquaculture with emphasis on fish detection and behavior analysis: a review. *Archives of Computational Methods in Engineering*10.1007/s11831-020-09486-2 (2021).

[14] Weinstein, B. G. A computer vision for animal ecology. *J. Anim. Ecol.* **87**, 533–545. 10.1111/1365-2656.12780 (2018). 29111567 10.1111/1365-2656.12780

[15] Dang, J., Zhong, Y. & Qin, X. PPformer: Using pixel-wise and patch-wise cross-attention for low-light image enhancement. *Comput. Vis. Image Underst.* **241**, 103930. 10.1016/j.cviu.2024.103930 (2024).

[16] Qin, X. et al. Fourier boundary features network with wider catchers for glass segmentation. *IEEE Trans. Image Process.*10.1109/TIP.2025.3592522 (2025). 40737147 10.1109/TIP.2025.3592522

[17] Li, T. et al. SAM-Guided Semantic Knowledge Fusion for Visible-Infrared Object Detection. *Proceedings of the 33rd ACM International Conference on Multimedia*, 8835–8844 (2025). 10.1145/3746027.3755718

[18] Guo, Z. et al. Automatic detection of feral pigeons in urban environments using deep learning. *Animals* **14**, 159. 10.3390/ani14010159 (2024). 38200890 10.3390/ani14010159 PMC10777961

[19] Takeki, A. et al. Combining deep features for object detection at various scales: finding small birds in landscape images. *IPSJ transactions on computer vision and applications* 5. 10.1186/s41074-016-0006-z (2016).

[20] Jo, J., Park, J., Han, J., Lee, M. & Smith, A. H. Dynamic bird detection using image processing and neural network. *7th International Conference on Robot Intelligence Technology and Applications (RiTA)*, 210–214 (2019)., 210–214 (2019). (2019). 10.1109/RITAPP.2019.8932891

[21] Redmon, J., Divvala, S., Girshick, R. & Farhadi, A. You only look once: Unified, real-time object detection. *Proceedings of the IEEE conference on computer vision and pattern recognition*, 779–788 (2016). 10.1109/CVPR.2016.91

[22] Hong, S.-J., Han, Y., Kim, S.-Y., Lee, A.-Y. & Kim, G. Application of deep-learning methods to bird detection using unmanned aerial vehicle imagery. *Sensors* **19**, 1651. 10.3390/s19071651 (2019). 30959913 10.3390/s19071651 PMC6479331

[23] Jiang, T., Zhao, J. & Wang, M. Bird detection on power transmission lines based on improved YOLOv7. *Appl. Sci.* **13**, 11940. 10.3390/app132111940 (2023).

[24] Lei, J. et al. Optimized small waterbird detection method using surveillance videos based on YOLOv7. *Animals* **13**, 1929. 10.3390/ani13121929 (2023). 37370439 10.3390/ani13121929 PMC10295383

[25] Chen, X. et al. An efficient method for monitoring birds based on object detection and multi-object tracking networks. *Animals* **13**, 1713. 10.3390/ani13101713 (2023). 37238144 10.3390/ani13101713 PMC10215751

[26] Haag, K. H., Lee, T. M. & Water, T. *Hydrology and ecology of freshwater wetlands in central Florida: a primer* (US Geological Survey, 2010).

[27] Zhang, X. et al. RFAConv: Innovating spatial attention and standard convolutional operation. *arXiv preprint arXiv:2304.03198.*10.48550/arXiv.2304.03198 (2023).

[28] Lau, K. W., Po, L.-M. & Rehman, Y. A. U. Large separable kernel attention: Rethinking the large kernel attention design in cnn. *Expert Syst. Appl.* **236**, 121352. 10.1016/j.eswa.2023.121352 (2024).

[29] Liu, S., Qi, L., Qin, H., Shi, J. & Jia, J. Path aggregation network for instance segmentation. *Proceedings of the IEEE conference on computer vision and pattern recognition*, 8759–8768 (2018). 10.1109/CVPR.2018.00913

[30] Lin, T. Y. et al. Feature pyramid networks for object detection. *Proceedings of the IEEE conference on computer vision and pattern recognition*, 2117–2125 (2017). 10.1109/CVPR.2017.106

[31] Tan, M., Pang, R., Le, Q. V. & Efficientdet Scalable and efficient object detection. *Proceedings of the IEEE/CVF conference on computer vision and pattern recognition*, 10781–10790 (2020). 10.1109/CVPR42600.2020.01079

[32] Wang, J. et al. Carafe: Content-aware reassembly of features. *Proceedings of the IEEE/CVF international conference on computer vision*, 3007–3016 (2019). 10.1109/ICCV.2019.00310

[33] Hu, J., Shen, L. & Sun, G. Squeeze-and-excitation networks. *Proceedings of the IEEE conference on computer vision and pattern recognition*, 7132–7141 (2018). 10.1109/CVPR.2018.00745

[34] Woo, S., Park, J., Lee, J. Y., Kweon, I. S. & Cbam Convolutional block attention module. *Proceedings of the European conference on computer vision (ECCV)*, 3–19 (2018). 10.1007/978-3-030-01234-2_1

[35] Chabot, D. & Francis, C. M. Computer-automated bird detection and counts in high‐resolution aerial images: A review. *J. Field Ornithol.* **87**, 343–359. 10.1111/jofo.12171 (2016).

[36] Mesquita, G. P., Rodríguez-Teijeiro, J. D., Wich, S. A. & Mulero-Pázmány, M. Measuring disturbance at swift breeding colonies due to the visual aspects of a drone: a quasi-experiment study. *Curr. Zool.* **67**, 157–163. 10.1093/cz/zoaa038 (2021). 33854533 10.1093/cz/zoaa038 PMC8026149

[37] Moll, J. et al. Radar-based Detection of Birds at Wind Turbine Installations: Results from a Field Study. *23rd International Microwave and Radar Conference (MIKON)*, 285–288 (2020)., 285–288 (2020). (2020). 10.23919/MIKON48703.2020.9253826

[38] Phillips, A. C. et al. Efficacy of avian radar systems for tracking birds on the airfield of a large international airport. *Wildl. Soc. Bull.* **42**, 467–477. 10.1002/wsb.910 (2018).

[39] Creswell, A. et al. Generative adversarial networks: An overview. *IEEE. Signal. Process. Mag.* **35**, 53–65. 10.1109/MSP.2017.2765202 (2018).

[40] Xu, Z., Li, J. & Zhang, M. A surveillance video real-time analysis system based on edge-cloud and fl-yolo cooperation in coal mine. *IEEE Access.* **9**, 68482–68497. 10.1109/ACCESS.2021.3077499 (2021).

[41] Feng, H., Mu, G., Zhong, S., Zhang, P. & Yuan, T. Benchmark analysis of yolo performance on edge intelligence devices. *Cryptography* **6**, 16. 10.3390/cryptography6020016 (2022).

